# Does equipoise exist for masking children for COVID-19?

**DOI:** 10.1016/j.puhip.2023.100428

**Published:** 2023-09-09

**Authors:** Tracy Beth Høeg, Sebastián González-Dambrauskas, Vinay Prasad

**Affiliations:** a Department of Epidemiology and Biostatistics, University of California-San Francisco, USA; b Department of Clinical Research, University of Southern Denmark, Odense, Denmark; c Red Colaborativa Pediátrica de Latinoamérica (LARed Network), Montevideo, Uruguay; d Departamento de Pediatría y Unidad de Cuidados Intensivos de Niños del Centro Hospitalario Pereira Rossell, Facultad de Medicina, Universidad de la República, Montevideo, Uruguay

## Abstract

Clinical equipoise is characterized by genuine uncertainty within the medical community about the effectiveness of a medical intervention. Its existence is often deemed necessary for clinical trials and signals a need for higher quality evidence, most often with randomized controlled trials, before the intervention can be considered effective. A leading official of the United States' Centers for Disease Control and Prevention Director, when testifying before Congress in February of 2023, indicated there was no need for randomized controlled trials of masking because, owing to overwhelming evidence of benefit, there was no longer equipoise about masking children for COVID-19. We disagree with this statement and outline the reasons why in this piece. We review the concept of clinical equipoise specifically using the example of child masking. We list reasons equipoise still exists for masking children, including a lack of consensus among experts, contradictory medical evidence and recent and ongoing randomized efforts. Finally, we differentiate between clinical equipoise and ethical appropriateness. Despite ongoing equipoise about masking children, we outline why, owing to lack of evidence of net benefit, recommending this intervention does not currently appear to be medically ethical.

## 1 Introduction

On Feb 8, 2023, a leading official of the Centers for Disease Control and Prevention (CDC) testified before Congress and was asked why the agency did not perform any randomized controlled trials of masking, specifically with respect to children [1]. The CDC official replied, “I'm not sure anybody would have proposed a clinical trial because, in fact, there wasn't equipoise to the question anymore,” and then alluded to a number of observational studies that had suggested evidence of benefit.

The position that equipoise does not exist for masking children for COVID-19 is contradicted by threer lines of evidence: 1. Disagreement among experts and variations in guidelines, 2. Ambiguity of evidence and 3. The presence of recent and ongoing randomized efforts.

We review the concept of equipoise, describe how equipoise appears in real life and at what point it may no longer exist. Finally, we discuss how, even if there is equipoise for masking children, most would consider it medically unethical to recommend any intervention when the totality of evidence fails to find a net benefit.

### 1.1 The history of clinical equipoise

The use of randomized trials to assess medical interventions dates back to a 1940s trial of streptomycin for tuberculosis [2]. At this time, the determination that there was sufficient uncertainty to warrant a randomized trial arose from individual clinicians having no treatment preference. This lack of treatment preference by an individual was termed “theoretical equipoise” by Benjamin Freedman in 1987 [3]. He argued a true lack of preference on the part of the investigator occurs so rarely that it would inappropriately preclude most trials [3]. For this reason, he proposed the broader “clinical equipoise,” defined as “genuine uncertainty within the medical expert community … about the preferred treatment.” [3] Though there have been critics of Freedman's “clinical equipoise,” his argument that uncertainty within the medical community is a more appropriate prerequisite for clinical trials than an individual lack of preference has become generally accepted, particularly as we discover many interventions clinicians strongly *believed* worked went on to be found ineffective in randomized studies [4].

### 1.2 Disagreement among experts and evidence of ongoing equipoise

Beliefs about the effectiveness and appropriateness of mask-wearing for respiratory infections vary widely by geographic location, type of mask, age and circumstance. The U.S. CDC, as of September 2023, continues to recommend [5] that children as young as 2 years old wear a high-quality mask or respirator when their community COVID-19 disease burden is considered “high” (or at “medium” disease levels if they themselves are considered “high risk”). This is in contrast to the European Centre for Disease Prevention and Control (ECDC) [6], which never recommended masking for COVID-19 for children under the age of 12. The World Health Organization never recommended masks for children under 6 and now only recommends masks in indoor situations where risk of exposure or severe disease is high [7]. CDC and ECDC specifically recommend higher quality medical or respirator masks, while the WHO indicates cloth masks are “acceptable.” [7] Thus there still appears to be equipoise about mask type. However, none of these international organizations recommend masking outdoors in non-crowded spaces, thus it seems there may no longer be equipoise for masking in this setting.

Multiple international experts have argued against masking children citing both a lack of high-quality evidence of benefit and concerns about harms to learning and development [[8], [9], [10]], especially among pre-school age children [10]. One review by physicians from Uruguay and the United States pointed to a long list of studies documenting harms associated with masking children including increased anxiety, physical discomfort, decreased learning ability and recognition of emotion and sound [11]. On the other hand, some experts from the United States [12,13] have pointed to the substantial effectiveness of masks noted in some observational studies against SARS-CoV-2 transmission as evidence masking children may even play a role in reducing systemic racism [13].

However, practically speaking, fewer and fewer people are wearing masks, even in most healthcare settings. This suggests there is a growing consensus about masking among the general public. At the same time, in the summer and fall of 2023, a number of educational programs [14,[15], [31]]continue to require masks for children under certain circumstances based on the CDC's guidance. It is unclear if more masking requirements may return to schools over the coming winter months or for other respiratory infections. The different viewpoints heldby medical experts indicates equipoise continues to exist for masking children even down to the age of two.

### 1.3 Ambiguity of the current evidence

A fundamental reason for the continued lack of consensus about mask-wearing for respiratory viruses is ambiguity of evidence. However, data from the two existing Cochrane Reviews of randomized data have been consistent, and unsupportive of masking. These reviews published in 2020 and 2023 [16,17], included randomized trials of surgical/medical masks and N95/P2 respirators, with some study participants as young as five. The first included 14 trials for influenza, influenza-like illness and respiratory syncytial virus in the community, healthcare and home settings. It concluded that masks did not result in a clear reduction of disease, although there was low to moderate certainty in their conclusions. The follow up review included 17 trails with 3 randomized trials for COVID-19 and, again, pooled results regarding medical or surgical masks compared with no masks concluded “wearing masks in the community probably makes little or no difference to the outcome of laboratory‐confirmed influenza/SARS‐CoV‐2 compared to not wearing masks.*”* The authors stated data were “very uncertain” about N95/P2 respirators.

### 1.4 Contradictory observational studies

Numerous observational studies conducted during the COVID-19 pandemic found mask wearing to be associated with lower case rates [13,[18], [19], [20]]. However, given places and people who wear masks tend to differ in many ways beyond mask-wearing, these studies face substantial, if not insurmountable, challenges when attempting to adjust for confounding variables [21,22]. Many studies did not include control or comparator groups [[23], [24], [25]]. Some associations between mask requirements and lower case rates may also be spurious due to limited study time frame or small population [22,26].

Observational studies are designed to look for an association between masking and lower case rates, but are, with very few exceptions, unable to infer causality. Some natural experiments can also substantially reduce confounding by choosing a situation where the only meaningful variable that differs is mask use. This appears to have been the case with a regression discontinuity study from Catalonia, Spain [27], which took advantage of 5- and 6-year-old children having differing mask policies [27]. Researchers found no significant difference in cases or transmission rates between the masked 6-year-olds and unmasked 5-year-olds.

However, other natural experiments, where a mask mandate disappears for one group but not another may face the challenge of not being able to adjust for confounding factors that change along with the mandate [13,22]. Randomized controlled trials are able to greatly reduce bias and are, assuming proper study design, much more reliable for ruling in or out specific causal relationships. However, these have not been conducted in all settings, including educational settings or limited to brief hospital encounters where N95/respirator masks are worn consistently. Ongoing disagreement in these circumstances likely stems from a lack of more certain evidence and is consistent with ongoing equipoise.

### 1.5 Recent and ongoing randomized investigations

The presence of numerous recent randomized trials of masking in the community and healthcare setting speaks to the fact that multiple independent expert groups simultaneously assessed the landscape of evidence and found it also compatible with equipoise. Randomized studies of masking were recently completed in Denmark, Bangladesh, Canada, Egypt, Israel, Pakistan, Guinea Bissau and there is one ongoing randomized study of masking in Norway. Notably, this ongoing study is not recruiting anyone younger than 18, thus will not provide specific data for children.

### 1.6 Masking children: medical ethics and the end of equipoise

Most of the world's population has immunity to COVID-19 and the severity of the disease has decreased drastically [28]. One study from the UK reported no omicron deaths in children who had already been infected [29], compared with an initial worldwide infection fatality rate in children of around 3/million [30]. Worldwide, adults in general are choosing not to mask. Thus, the question arises: Is there still *genuine uncertainty* about masking children for COVID-19. In other words, is there still equipoise?

As late as September of 2023, a group of experts and the US CDC [5] continues to recommend masking children two and older in certain circumstances. The ECDC and WHO continue to mention masking children over ages 11 and 5, respectively, as an option for disease mitigation [5,6]. Thus there appears to still be equipoise about masking children.

However, a careful discernment of the evidence reveals a lack of evidence of net benefit of this intervention. Thus ethically, according to the principle of non-maleficence, the intervention would be considered unethical. As public awareness increases about the absence of high-quality data demonstrating benefit, equipoise may disappear, though may once again reappear with the emergence or resurgence of another respiratory disease threat. At that time, it will be indicated to obtain high-quality evidence from randomized trials before concluding based on low-quality evidence that the benefits of masking children will outweigh the harms, even for a limited period of time.

## 2 Conclusion

A leading CDC official stated no randomized trials of masking were done in children due to a lack of equipoise, citing overwhelming benefits found in observational studies. However, the presence of widespread disagreement among experts, remaining ambiguity of evidence, with pooled randomized trials being negative, and the presence of recent and ongoing randomized investigations all support the presence of equipoise. At the same time, weighing the current high-quality evidence with known and potential harms [11], recommending masking for children goes against basic medical ethics. Currently, the onus lies with the public health agencies that continue to recommend masking children, especially when this can lead to mandates, to produce high-quality data to guide their recommendations rather than rely on low-quality observational data as if it were settled science.

## Funding

None.

## Declaration of competing interest

The authors declare the following financial interests/personal relationships which may be considered as potential competing interests: Vinay Prasad's Disclosures. (Research funding) Arnold Ventures (Royalties) Johns Hopkins Press, Medscape, and MedPage (Honoraria) Grand Rounds/lectures from universities, medical centers, non-profits, and professional societies. (Consulting) UnitedHealthcare and OptumRX. (Other) Plenary Session podcast has Patreon backers, YouTube, and Substack. Tracy Beth Høeg's Disclosures. (Honoraria) Brownstone Institute, Global Liberty Institute. (Consulting) Florida Department of Health (Other) Substack, payment for writing at The Atlantic, Los Angeles Times, New York Times, The Hill and Tablet Magazine. Dr. González has no disclosures to report.
